# Type D Personality and Alexithymia: Common Characteristics of Two Different Constructs. Implications for Research and Clinical Practice

**DOI:** 10.3389/fpsyg.2018.00106

**Published:** 2018-02-09

**Authors:** Maria S. Epifanio, Sonia Ingoglia, Pietro Alfano, Gianluca Lo Coco, Sabina La Grutta

**Affiliations:** ^1^Department of Psychology and Educational Sciences, University of Palermo, Palermo, Italy; ^2^Consiglio Nazionale delle Ricerche, Institute of Biomedicine and Molecular Immunology, Rome, Italy

**Keywords:** alexithymia, type-D personality, psychosocial risk factors, TAS-20, DS-14

## Abstract

In the last few decades, particular attention has been paid to the role of personality specific traits that can affect the loss of health, i.e., Type D personality and Alexithymia. They have been conceptualized in a different period, this means that they are different both for their theoretical positions and their empirical studies. Some authors have speculated that there is a potential conceptual overlap between Type D personality and alexithymia constructs but there is a shortcoming in the literature. The aim of the study was to examine the potential overlap between the constructs of type D personality and alexithymia, replicating previous two studies, to extend these findings to Italian population. The participants were 247 Italian adults (males = 43%), recruited in primary health care practices of Palermo. All participants did not have chronic diseases during tests administration. They ranged in age from 35 to 69 years old (*M* = 52.34 years, *SD* = 9.76). Participants were administered Type D Personality Scale (DS-14) and Toronto Alexithymia Scale (TAS-20). A series of confirmatory factor analyses was performed to evaluate the factorial structure underlying the TAS-20 and DS-14 items. Globally results showed that alexithymia and type D personality are distinct constructs, but they are also strictly positively related with each other. Negative affectivity (NA) was highly correlated with Difficulties in identifying feelings and Difficulties in describing feelings, while Social inhibition (SI) was highly correlated with Difficulties in describing feelings. These results are consistent with those of other studies conducted in this area. Future research should consider evaluating the relationship between a deficit of affect regulation and type D personality to improve the effectiveness of interventions of health cure.

## Introduction

Research has extensively supported the role of psychological risk factors both in the pathogenesis and outcomes of physical diseases among different patient populations (Lumley et al., 1997, 2007; Taylor et al., 1997; Kudielka et al., 2004; Porcelli, 2009; Basinska and Wozniewicz, 2013; Solano, 2013). These studies have shown associations between individual's personality dimensions and a self-management of physical disease. In the last few decades, a particular attention has been paid to the role of personality specific traits (i.e., Type D personality and Alexithymia) which can affect the loss of health. They have been conceptualized in a different period, this means that they are different both for their theoretical positions and their empirical studies. By the contrast, it seems to be a potential conceptual overlap between the constructs.

In 1995, Denollet and colleagues introduced the construct of Type-D personality. It is defined such as the subject's general tendency to psychological distress, characterized by negative affectivity (NA) and social inhibition (SI) (Denollet et al., 1995). NA is concerned with tendencies to experience negative emotions such as dysphoria, depressive mood, anxiety, hostility, anger, and irritability, whereas SI is expressed in a tendency to avoid the expression of these negative emotions as well as the behaviors associated with these dysfunctional emotions (Denollet and Conraads, 2011; Gremigni and Casu, 2013). Inhibition occurs mostly in social situations, and the individual is aware of being inhibited. The risk factor would be the synergy between these two dimensions (NA and SI) and not by single factors. It is worth noting that this personality construct emphasizes the normal characteristics of personality more than psychopathological aspects. However, individuals with high levels on both traits (NA and SI) are more likely to experience chronic distress (Mols and Denollet, 2010).

Interestingly, the first ideas about the Type D personality were not derived from a theoretical model, but rather emerged from empirical analyses which aimed to demonstrate the negative effect of repressive coping (Denollet et al., 1995; Grande et al., 2013). For example, Denollet linked emotional distress to stable personality traits but a comprehensive theoretical model of the pathogenic mechanisms that operate through the interaction of NA and SI has not been developed (Grande et al., 2013).

Type D personality was originally developed to understand the role of specific psychological factors in the outcomes among cardiovascular patients' population (Pedersen and Denollet, 2003). Recently it has been increased an interest in the construct and its value is confirmed in several patient's population (Mols and Denollet, 2010). Recent meta-analytic results showed that Type D is associated with poor mental and physical health status both in clinical and non-clinical populations (Mols and Denollet, 2010). This type of personality ranges from 13 to 32.5% in the general population and from 26 to 35% in patients affected by cardiovascular disease. Type D personality it is considered one of the psychopathological conditions that affect health and longevity, therefore, it entails psychological and medical treatment (Kheradmand et al., 2016). Moreover, people with type D personality are at the continuous risks of psychiatric and physical disorders (Ogrodniczuk et al., 2012; Basinska and Wozniewicz, 2013; van Middendorp et al., 2016).

In 2005 Denollet constructed the Type-D Scale (DS-14), a scale that contains two sub-scales, namely Negative Affective and Social Inhibition which are scored in a Likert scale. It is the instrument largely employed to measure the Type D personality. Several cross-cultural studies carried out in Europe both in cardiovascular patient populations and in general population confirm the validity and reliability of DS-14 scale (Pedersen and Denollet, 2003; Denollet, 2005). The DS-14 not only can be evaluated in cardiovascular population but also in other clinical and general populations (Emons et al., 2007; Spindler et al., 2009; Grande et al., 2010; Howard and Hughes, 2012; Kupper et al., 2013), as well as chronic pain (Barnett et al., 2009). Gremigni and Sommaruga (2005) highlighted the good psychometric properties of the Italian version of DS-14, and they recommend its use in psychological screening for rehabilitation and clinical research.

The Alexithymia is a personality construct which refers to one's inability to successfully deal with emotional regulation (Taylor and Bagby, 2000). This construct consists of the following components: difficulty in identifying and describing feelings, an impoverished fantasy life, and externally-oriented thinking (Taylor et al., 1991).

Alexithymia is usually defined such as a deficit in the mental processing of feelings and emotional experiences (affect regulation), that produced a bounded ability to express feelings and a widely developing about the emotional experience. Indeed, the individuals affected by alexithymia find complication distinguishing specific emotions, identifying feelings from body sensations and possess an externally orientated way of thinking (Sifneos et al., 1977). They are often assailed by a widespread negative affection, social evasion and poor emotionally relationships with other people.

In the early 1970's Nemiah and Sifneos (1970) introduced the construct (Sifneos, 1973; Nemiah et al., 1976). Originally, their study was based on several clinical observations about the cognitive and affective style of patients with classic psychosomatic disease but Alexithymia has been found to be associated with poor health outcomes in a variety of populations (Kauhanen et al., 1994, 1996; Valkamoa et al., 2001; Taylor and Bagby, 2004; Henry et al., 2006; Lumley et al., 2007).

Over 40 years after the original definition, a great deal of evidence supports the connection between difficulties with affect regulation and poor physical and mental health. Alexithymia it is now widely recognized as a trans-nosographic construct, that is as a non-specific risk factor for many physical diseases such as neoplastic diseases as breast cancer, chronic pain syndrome, essential hypertension, chronic urticaria (Taylor et al., 1997; Epifanio et al., 2005a,b, 2013; Maniaci et al., 2006; Porcelli, 2009), functional gastrointestinal disorders, and for many mental disease such as depression, eating disorders, addiction disorder, dissociative disorders, Post-traumatic Stress Disorder, panic attacks (Caretti and La Barbera, 2005; Taylor and Bagby, 2013; Epifanio et al., 2014).

The TAS-20 (Bagby et al., 1994) is the instrument largely employed to its measure. TAS-20 assesses the presence of alexithymic characteristics by three factors: difficulty in distinguishing between feelings and emotions, difficulty in identifying and describing feelings, externally-oriented thinking. Thanks to good psychometric characteristics, TAS-20 is a measure widely used. This has allowed both the comparison as well as the generalizing the results of adults subjects mostly.

Several studies underlined common characteristics between Type-D and Alexithymia constructs: both dimensions of Type D (i.e., NA and SI) and Alexithymia were positively correlated with neuroticism and negatively correlated with extroversion in general population (De Fruyt and Denollet, 2002; Yekta et al., 2011) and they were associated with anxiety and depression (Kudielka et al., 2004; Schiffer et al., 2008; Tselebis et al., 2010; Korkoliakou et al., 2014; Nekouei et al., 2014). Both in type D and alexithymic individuals there is a predominance of an insecure attachment style which is, for its part, associated with a deficit of affective regulation (Huis in't Veld et al., 2011). Both constructs are only some of the crucial risk factors for cardiovascular diseases that encourage unhealthy lifestyles; they can also be defined as non-specific risk factors (Mols and Denollet, 2010; Epifanio et al., 2014). However, they can be found in other clinical and general populations.

According to these studies, some authors have speculated a potential conceptual overlap between Type D personality and alexithymia constructs but there is still a dearth of research on this topic. Only two previous studies have examined the link between Type D personality and Alexithymia simultaneously in a Scottish students' sample (Williams et al., 2011) and in Iranian students' sample (Kheradmand et al., 2016).

Williams et al. (2011) highlighted that the Type D personality and Alexithymia are conceptually and theoretically overlapping constructs but at the same time they show some distinct factor structures. A series of confirmatory factor analyses was performed and results showed that alexithymia and type D personality are distinct constructs, but they are also strongly related to each other. This study should be considered as preliminary within this line of research, but it is limited since the sample was composed predominantly by female university students. The authors underlined the need to conduct further research on clinical and non-clinical samples to better understand the relationship which occurs among these two constructs.

Kheradmand et al. (2016) investigated the “simultaneous” factor structure of Alexithymia and type D personality, reproducing the same research method used by Williams et al. (2011) in a sample of Iranians students. The results confirmed previous data and conclusion: alexithymia and Type-D personality are overlapping but they also are distinct constructs.

## The present study

**Figure 1:**
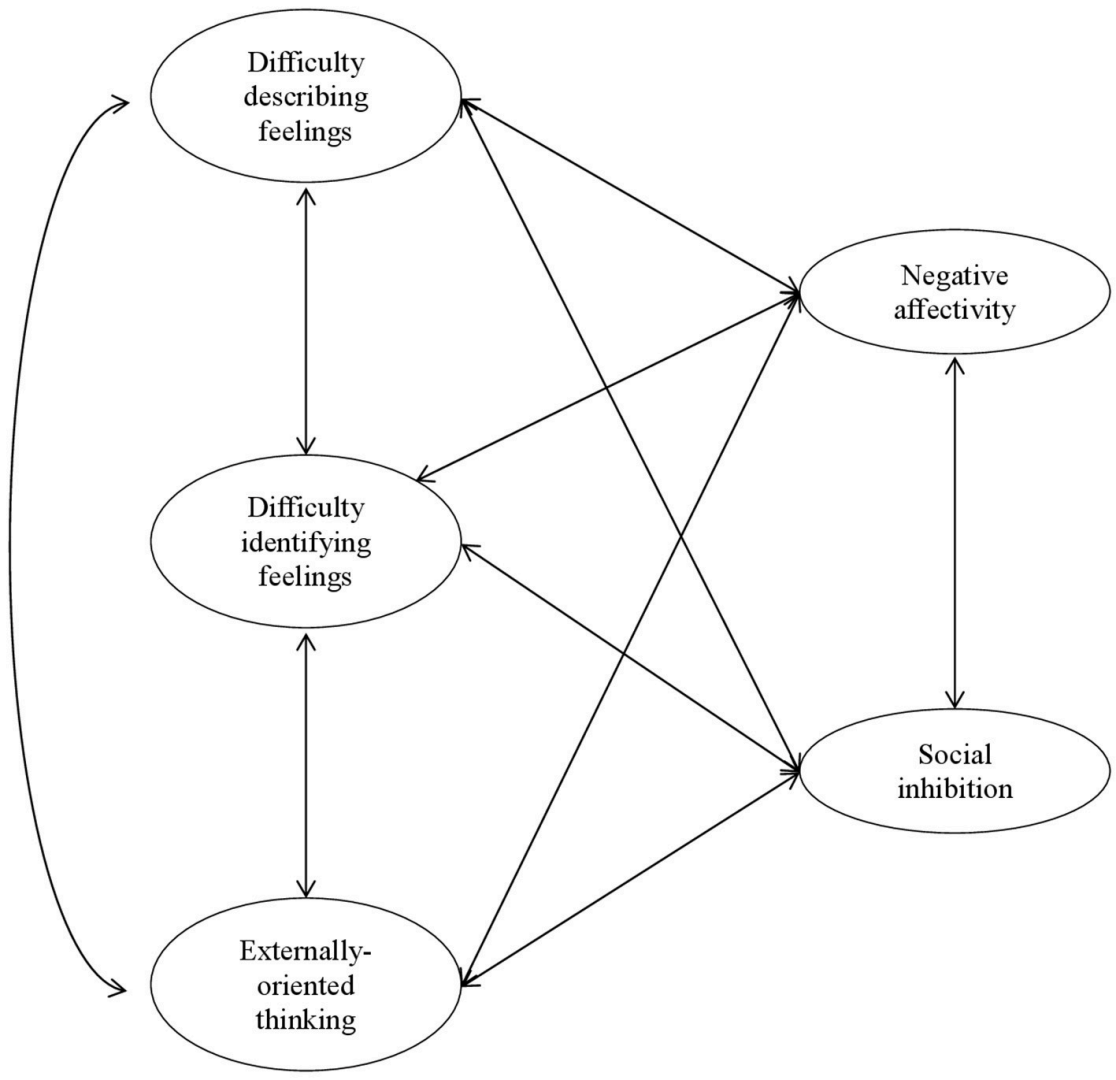
Hypothesized model of the relations between alexythimia and type D personality.

The aim of the study was to examine the potential overlap between the constructs of type D personality and alexithymia, replicating the studies by Williams et al. (2011) and Kheradmand et al. (2016). The hypothesized model of the relations between the constructs appears in Figure 1. Moreover, the current study was also aimed at extending these findings to a different sample. Our sample was composed by Italian adults who were older than Scottish and Iranian university students' sample and with different socio-cultural characteristics. We believe that this is necessary in order to extend the knowledge and to increase the understanding of these constructs which are used both in clinical activities and in the treatment of patients with physical illnesses.

## Materials and methods

### Participants

This research was part of a larger research program designed to explore the relationship between Alexithymia, Type-D personality and cardiovascular risk in the non-clinical population. For the purpose of the current study, 277 consecutive Italian adults routinely attending medical check-ups from their physician, were recruited in primary health care practices in the province of Palermo. Information on clinical characteristics was obtained from the patients' medical records and included diabetes, hypertension, hypercholesterolemia, angina, left ventricular ejection fraction (LVEF), ischemic heart disease (IHD), or congestive heart failure (CHF). Thirty participants were excluded from participation in the study because they had a diagnosis of these diseases. The 247 remaining participants were individuals belonging to a non-clinical population. All participants received written information about the study and written informed consent was obtained from them.

The 247 participants (males = 43%) ranged in age from 35 to 69 years old (*M* = 52.34 years, *SD* = 9.76). Thirteen percentage of the participants were unmarried, 80% were married, 5% were separated or divorced, and 2% were widow/widower. With regard to their educational status, 20% were university graduated, 39% had obtained a certificate of secondary education, and 41% had eight or less years of education. With regard to their occupational status, 55% were workers, 26% were housewives, 12% were retired, and 6% were unoccupied.

### Measures

#### Alexithymia

Participants were administered the Toronto Alexithymia Scale (TAS-20; Taylor et al., 1992; Italian adaptation by Bressi et al., 1996). It consists of 20 items articulated in three subscales: Difficulty identifying feelings (DIF), which evaluates the difficulty in recognizing, feelings and distinguish emotions from feelings (5 items, e.g., “I am often confused about what emotion I am feeling”); Difficulty describing feelings to others (DDF), which evaluates the difficulty in verbalizing feelings to others (5 items, e.g., “It is difficult for me to find the right words for my feelings”); Externally oriented thinking (EOT), which evaluates the tendency of individuals to focus their attention externally and to use concrete way of thinking (8 items, e.g., “Looking for hidden meaning in movies or plays distracts from their enjoyment”). Items were scored on a 5-point Likert scale ranging from 1 (*strongly disagree*) to 5 (*completely agree*). A total score higher than 60 characterizes individuals with Alexithymia. In the present study, the scale had adequate internal consistency: Cronbach's alpha coefficients were 0.88, 0.82, and 0.78 for DIF, DDF, and EOT, respectively.

#### Type D personlality

Participants were administered the Type-D Scale 14 (DS14; Denollet, 2005; Italian adaptation by Gremigni and Sommaruga, 2005). It consists of 14 items articulated in two subscales: NA, which evaluates dysphoria, anxiety and irritability (7 items, e.g., “I am often down in the dumps”); SI, which evaluates social discomfort, reticence and lack of social poise (7 items, e.g., “I often feel inhibited in social interactions”). Items were scored on a 5-point Likert scale ranging from 0 (*false*) to 4 (*true*). A score higher than 10 in both subscales characterizes individuals with a type D personality. In the present study, the scale had adequate internal consistency: Cronbach's alpha coefficients were 0.86 for NA and 0.83 for SI.

#### Procedure

The Institutional Review Boards (IRB) of the University of [blinded for the review process] approved this study. It was conducted in conformity with the guidelines provided by the Italian Association of Psychology (Associazione Italiana di Psicologia, 2014) for the ethical treatment of the participants. All participants voluntarily agreed to take part in this investigation without receiving compensation. A prior permission was obtained from each of them. Participants were consecutively recruited at health primary care service of Palermo from 2015 to 2016. Several trained psychologists administered DS-14 and TAS-20 in doctor's waiting rooms. All participants received written information about the study and the scales administration did not take more than 20 min to complete.

#### Data analysis approach

A series of Confirmatory Factor Analyses (CFA) was performed to evaluate the associations between type D personality and alexithymia. The hypothesized model appears in Figure 1. The CFA was based on examining the covariance matrix using Mplus 7 software (Muthén and Muthén, 1998-2012). In order to establish the measurement scale of each factor, their variance was fixed to 1. Since the items exhibited a multivariate non-normal distribution (the normalized Mardia's coefficient was 8.18, *p* < 0.001), the robust maximum likelihood (MLR) estimation method was used. It adjusts standard errors of parameter estimates and chi-square statistics (SBχ^2^) to account for non-normality (Satorra and Bentler, 1994). The goodness of fit of the model was assessed using a range of goodness-of-fit statistics and evaluation of the appropriateness of the model parameters. The χ^2^ statistic assessed the sample and implied covariance matrix with a good-fit model being indicated by a non-significant result. However, the χ^2^ statistic is strongly associated with sample size, and as such, good models tend to be excessively rejected. Therefore, Tanaka (1987) suggested that a model should not be rejected simply on the basis of a significant χ^2^ result. Therefore, model fit was judged to be good if Comparative Fit Index (CFI; Bentler, 1990) and Tucker–Lewis Index (TLI; Tucker and Lewis, 1973; Bentler and Bonett, 1980) ≥0.95, if Root Mean Square Error of Approximation (RMSEA; Steiger, 1990) < 0.05, and Standardized Root Mean Squared Residuals (SRMR; Jöreskog and Sörbom, 1993; Hu and Bentler, 1999) < 0.5.

Given the small size of the sample with respect to the number of observed variables, the number of indicators of the factorial model was reduced by using item parceling (Bandalos and Finney, 2001). Three item parcels were built for each latent dimension, thus ending with 15 parcels (each parcel contained 2–3 items). Following Hattie (1985) to test the unidimensionality of each parcel (Bandalos and Finney, 2001), we examined the SRMR associated to a one-factor solution derived from a ML Exploratory Factor Analysis. SRMRs of the 15 parcels ranged from 0.00 to 0.06 (*M* = 0.032, *SD* = 0.015). Thus, we considered unidimensionality achieved for all parcels.

## Results

### Preliminary analyses

In order to explore how some personal characteristics may influence the levels of alexithymia and the presence of Type-D personality, a series of chi square analyses was performed. Results revealed the existence of differences related with educational level for both alexithymia [$\chi_{\left(2\right)}^{2}$ = 31.50, *p* < 0.001] and Type D personality [$\chi_{\left(1\right)}^{2}$ = 11.76, *p* = 0.001]; more specifically, 70% of participants with a low educational level tended to be classified as alexithymic and 66% of them tended to be classified with a Type D personality. Results revealed no significant associations of alexithymia and Type-D personality with gender [$\chi_{\left(2\right)}^{2}$ = 2.79 ns, for alexithymia, $\chi_{\left(1\right)}^{2}$ = 0.51 ns, for Type-D personality], and marital status [$\chi_{\left(2\right)}^{2}$ = 0.12 ns, for alexithymia, $\chi_{\left(1\right)}^{2}$ = 2.89 ns, for Type-D personality].

### Descriptive statistics and correlations

Mean, standard deviation, skewness, kurtosis, and Pearson's correlation coefficients of the parcels for study variables are given in Table 1. The data had a normal univariate distribution, skewness, and kurtosis values being approximately in the range −1 and +1 (Muthén and Kaplan, 1985) (Table 1).

**Table 1:** Means and standard deviations of study variables.

	**NA1**	**NA2**	**NA3**	**SI1**	**SI2**	**SI3**	**DDF1**	**DDF2**	**DDF3**	**DIF1**	**DIF2**	**DIF3**	**EOT1**	**EOT2**	**EOT3**
NA1	–														
NA2	0.627	–													
NA3	0.564	0.510	–												
SI1	0.248	0.373	0.312	–											
SI2	0.310	0.395	0.333	0.466	–										
SI3	0.324	0.423	0.489	0.462	0.558	–									
DDF1	0.240	0.150	0.268	0.331	0.281	0.315	–								
DDF2	0.157	0.175	0.271	0.168	0.234	0.311	0.315	–							
DDF3	0.177	0.146	0.192	0.173	0.205	0.286	0.177	0.293	–						
DIF1	0.496	0.397	0.547	0.247	0.265	0.378	0.380	0.339	0.178	–					
DIF2	0.457	0.329	0.505	0.180	0.213	0.304	0.329	0.366	0.249	0.638	–				
DIF3	0.407	0.367	0.458	0.182	0.263	0.250	0.364	0.320	0.241	0.546	0.655	–			
EOT1	0.095	0.122	0.161	0.226	0.147	0.174	0.385	0.139	0.125	0.152	0.129	0.113	–		
EOT2	0.158	0.196	0.200	0.230	0.200	0.329	0.259	0.138	0.221	0.186	0.159	0.199	0.305	–	
EOT3	0.119	0.126	0.072	0.164	0.168	0.243	0.271	0.091	0.164	0.074	0.101	0.117	0.258	0.338	–
*M*	1.82	1.22	2.10	1.55	1.00	1.11	2.51	2.50	3.02	2.41	2.06	2.24	2.42	2.99	2.50
*SD*	1.11	1.08	1.12	0.98	1.17	1.03	1.15	1.15	1.54	1.09	1.13	1.29	1.03	0.97	1.01

*NA, negative affectivity; SI, social inhibition; DDF, difficulty in describing feelings; DIF, difficulty in identifying feelings; EOT, externally-oriented thinking. Coefficients higher, in absolute value, than 0.15 were significant at p < 0.05*.

### Confirmatory factor analyses

**Figure 2:**
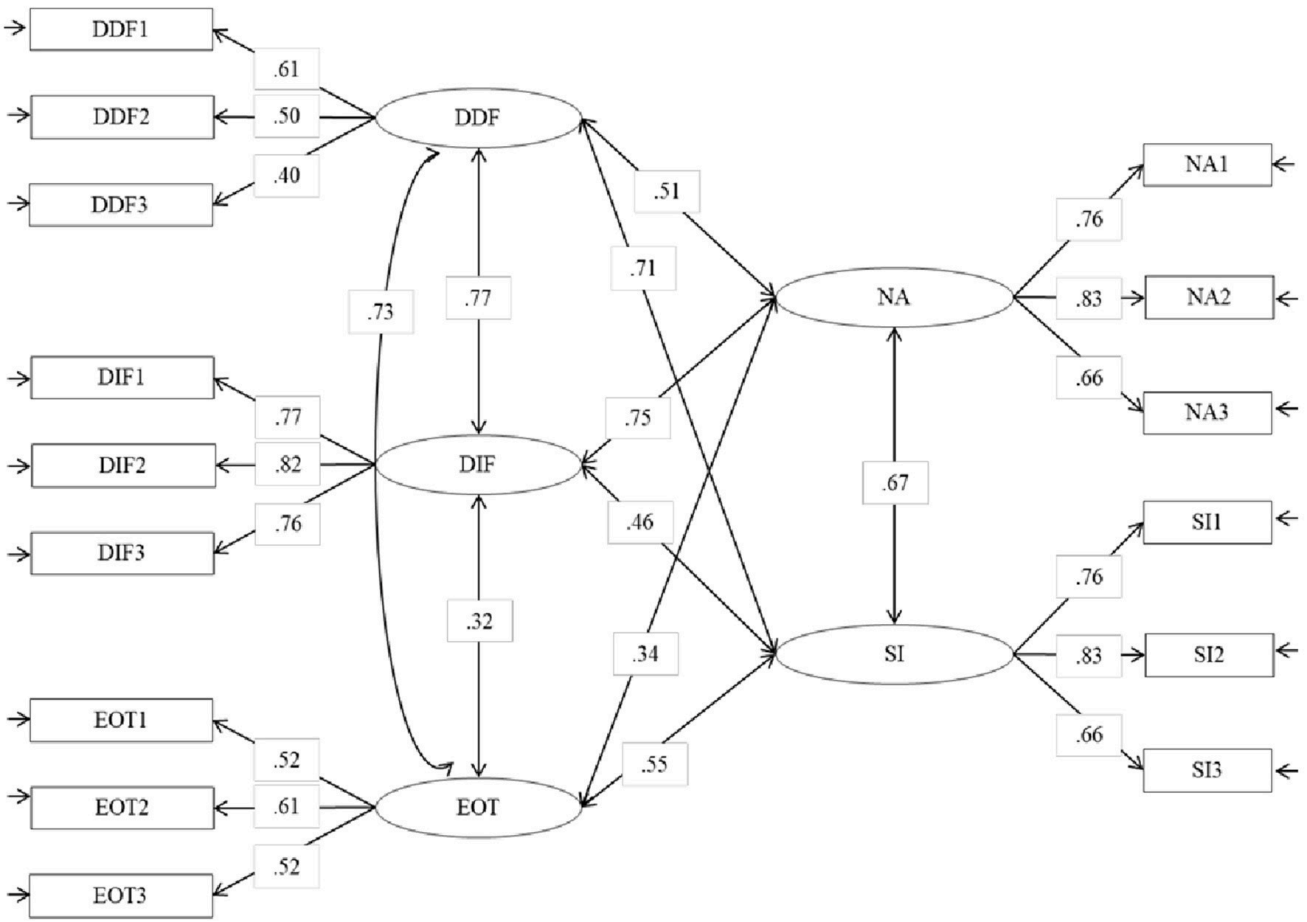
Statistical model of the relations between alexythimia and type D personality. Standardized solution, all parameters are significant with *p* < 0.05.

A series of CFA was performed to evaluate the interrelations between type D personality and alexithymia dimensions as measured by DS14 and TAS20, respectively. Goodness-of-fit indices for alternative models being run are shown in Table 2. Firstly, a one-dimensional model was tested. It did not fit the data very well. The best fitting model was a five-correlated factors model: 3 factors were related to the TAS-20 subscales (DIF, DDF, and EOT), and 2 factors were related with the DS-14 subscales (NA and SI). The standardized solution is shown in Figure 2. Results showed that NA was highly correlated with DIF, while SI was highly correlated with DDF.

**Table 2:** Goodness-of-fit indexes for alternative CFA models.

	**SBχ^2^**	**df**	***p***	**CFI**	**RMSEA**	**RMSEA 90% C.I**.
One-factor model	338.48	90	<0.001	0.774	0.106	0.094–0.118
Five-factors model	107.60	80	0.02	0.975	0.037	0.015–0.054

## Discussion

The aim of the study was to examine the potential overlap between the constructs of type D personality and Alexithymia, replicating the studies performed by Williams et al. (2011) and Kheradmand et al. (2016) with Scottish and Iranian university students, respectively. Authors found that alexithymia and type D personality are distinct constructs, but they are also strictly related to each other. Our study was also aimed at extending these findings to a different sample, composed by Italian adults recruited in primary health care practices but all participants did not have chronic diseases. They were adults, with an average age of fifty-two and the most of them were married.

The results of the present study confirmed previous findings showing that alexithymia and type D personality are generally distinct constructs. The dimensions DDF, DIF and EOT underlying TAS20 (the scale more largely used to assess alexithymia) were clearly differentiated from the dimensions NA and SI underlying DS14 (the scale more largely used to assess type D personality). Notwithstanding, some dimensions of alexithymia were highly correlated with some dimensions of type D personality. More specifically, NA was highly correlated with DIF and DDF, while SI was highly correlated with DDF. The deficit in the mental processing of feelings and emotional experiences (DIF and DDF) can produce a prevalence of NA such as dysphoria, depression, anxiety, anger, hostility, a tendency to refer mostly somatic symptoms and difficulties in the interpersonal relationship (SI). For example, individuals with a high level of alexithymia can have sudden outbursts of anger and/or tears but they do not know why. That is because the individual with alexithymic functioning has an emotional activation but he or she does not recognize the feeling associated because of a deficit in emotional processing. We believe that the NA could be considered such as an excessive level of express emotion, an affect dysregulation “upward.” Furthermore, individuals with a high level of alexithymia have also difficulties in the interpersonal relationship because they cannot put into words what they are experiencing, they are not able to communicate their feeling to others, and they are not able to establish an empathic relationship. The difficulties in emotional processing bring the individual with high levels of alexithymia to avoid interpersonal relationship. So, SI could be an expression of these difficulties.

Ogrodniczuk et al. (2012) examined the association between type D personality and alexithymia in a psychiatric outpatient sample. They found that patients with Type D Personality also presented high levels of alexithymia (more than individuals with Type non-D personality), large difficulty in describing emotions and thinking oriented to the outside. The authors explained the association between NA and DIF as that: “…type D persons may be aware of their negative emotions, their awareness may be more a vague perception of distress rather than a clear appreciation of the precise nature of the distressing emotions. Furthermore, SI associated with Difficulty Describing Feelings can suggest that the socially avoidant behavior of type D persons might be related to a difficulty in articulating their emotional experiences to others” (Ogrodniczuk et al., 2012, p. 129). If so, the Type D would be a stable tendency to cope emotions at the base of which there is the affective dis-regulation. Alexithymia could explicate the Type D personality structure. This suggest that is necessary to conduct further researches aimed at understanding the relationship between a deficit of affect regulation and type D personality. If that is the case, then it has important clinical implications: elevated levels of alexithymia may influence outcomes of both psychodynamic psychotherapy (Ogrodniczuk et al., 2011) and treatments of functional somatic disorders (Porcelli et al., 2003, 2007). It is widely acknowledged that assessment of alexithymia can inform treatment strategies and prognosis of various somatic disorders (Lumley et al., 2007; Taylor and Bagby, 2012). Therefore, only measuring the type D personality cannot be sufficient to promote the effectiveness of interventions of health care. Furthermore, when Type-D is linked to alexithymia, in this specific case, it is necessary to promote psychological intervention helping individuals to recognize, to verbalize and to become aware of feelings and only after this process the individual's emotional response can be modulate.

The use of both scales can increase our ability to spot some of the special condition that are strictly connected with the development or the exacerbation of somatic disease. Although there is evidence that some personality traits can trigger the onset of the somatic disease or interfere with the care, they may be also influenced by a disease which can be chronic or incurable. Hence, the previous cross-sectional studies with individuals who have already had the disease show some limits. For example, they were unable to establish specific relations between personality traits and chronic diseases. In some studies, the presence of type D personality varies over time in dialysis patients (Loosman et al., 2017) and in fibromyalgia patients (van Middendorp et al., 2016). Therefore, type D personality is possibly more a state instead of a trait phenomenon, such as the study carried on the fibromyalgia presumes.

Finally, our preliminary analyses showed a significant association of alexithymia and Type D personality with educational level; more specifically, we found that participants with low educational levels tended to report higher levels of alexithymia and Type D personality.

This finding is consistent with the few studies on Alexithymia and Type D personality conducted in general population to date (Mattila et al., 2010; Beutel et al., 2012). However, the link among these psychological constructs and some socio-demographic characteristics is still unclear, given the cross-sectional nature of previous research. One possible explanation of this result could be associated to the adoption of self-report scales and to individual's difficulty of understanding the text of the items that require a good educational level. Thus, further research is needed to examine this educational bias in a wide sample.

There are some limitations of the current study which should to be noted. First, the sample size is tiny, but it is focused on Italian adults who have different sociodemographic characteristics than samples in previous studies. Second, this study is also limited by the use of self-report measures. The self-report measures have some limitations, such as poor self-insight, dissemblance, and various style responses (Keefer et al., 2015). Particularly, the exclusive use of a self-report measure to assess alexithymia could be criticized because individuals with impaired affect awareness can accurately rate themselves on this lack of awareness on a self-report scale. For these reasons, it may be useful to examine the overlap of alexithymia and Type D using other measures, in addition to the TAS-20, i.e., the Toronto Structured Interview for Alexithymia (TSIA, Bagby et al., 2006). TSIA allowed bypassing the limitations associated with the self-report method for measuring Alexithymia (Keefer et al., 2017). This suggest that is necessary to conduct further researches for understanding the process of emotion regulation associated with type D to improve the effectiveness of interventions of health cure.

## Author contributions

MSE, SI, PA, GL, and SL contributed equally to the conception and design of the study as well as to the analysis and interpretation of data. MSE wrote the first draft of the manuscript and SI, GL, and SL made critical revisions. All authors approved the final version of the manuscript for publication.

### Conflict of interest statement

The authors declare that the research was conducted in the absence of any commercial or financial relationships that could be construed as a potential conflict of interest.
